# Prevalence of Metabolic Syndrome and its Related Factors in Bangladeshi Adults: A Cross‐Sectional Study

**DOI:** 10.1002/edm2.491

**Published:** 2024-06-09

**Authors:** Nurshad Ali, Abu Taher, Aporajita Das Trisha, Nusrat Jahan Koley, Khandaker Atkia Fariha, Farjana Islam

**Affiliations:** ^1^ Department of Biochemistry and Molecular Biology Shahjalal University of Science and Technology Sylhet Bangladesh; ^2^ Department of Geography and Environment Shahjalal University of Science and Technology Sylhet Bangladesh

**Keywords:** adults, Bangladesh, metabolic syndrome, prevalence, risk factors

## Abstract

**Objectives:**

Metabolic syndrome (MetS) is a group of medical conditions that elevate the chances of developing cardiovascular disease, stroke and Type 2 diabetes. This study aimed to determine the frequency and contributing risk factors of MetS in adults from Bangladesh.

**Methods:**

In this cross‐sectional study, 653 individuals (470 males and 183 females) were randomly selected to participate. Fasting blood samples were collected and analysed using standard methods to measure biochemical parameters. MetS was defined on the basis of NCEP‐ATP III guidelines, and multivariate logistic regression analyses were conducted to identify factors associated with MetS.

**Results:**

The prevalence of MetS was 19.7% in the healthy control group, 70.2% in the hypertensive group and 46.8% in the diabetic group. Overall, there was no significant difference in the prevalence of MetS between males (45%) and females (45.9%). The participants who had both hypertension and diabetes had the highest prevalence of MetS at 77.3%. Both males and females showed an increased trend in the prevalence of MetS and its components as they aged, except for WC in males (*p* < 0.01 for all cases). The 46–55 age group in males had a higher prevalence of MetS (68%), whereas the >55 age group in females had a prevalence of 73.9%. The most common component of MetS was low levels of HDL‐C, which affected over 80% of the studied sample. According to the logistic regression analyses, age, BMI, hypertension and diabetes were significantly associated with MetS in both genders.

**Conclusion:**

This study found a high prevalence of MetS in Bangladeshi adults. Several factors are significantly associated with the risk of MetS. It is crucial to consider the varying prevalence rates of MetS by age and gender as well as its different components while providing health guidance and support.

## Graphical abstract

Overall, there was no significant difference in the prevalence of MetS between males and females, but differences existed in the prevalence of MetS components between the two groups.

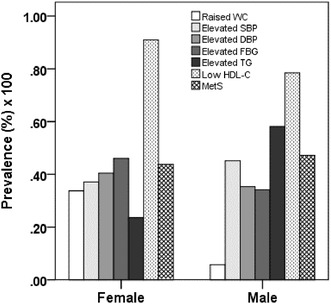

## 1 Introduction

Noncommunicable diseases (NCDs) are a significant public health concern in developing countries and the leading cause of mortality and disease burden worldwide. NCDs account for approximately 74% of all global deaths yearly, with 77% occurring in low‐ and middle‐income countries [1]. In addition to the unprecedented consequences of NCDs, there is also a growing challenge for global public health systems because of the increase in metabolic syndrome (MetS) cases. Patients with MetS have a significantly higher risk of developing NCDs, which poses a significant burden on public health [2]. MetS is a cluster of cardio‐metabolic abnormalities that include abdominal obesity, elevated blood pressure, hyperglycaemia, hypertriglyceridemia and reduced HDL‐cholesterol, and is strongly associated with the increased risk of developing Type 2 diabetes, cardiovascular diseases and all‐cause mortality [3]. The global prevalence of MetS is varied from 12.5% to 31.4% on the basis of the definition considered [4]. Research suggests that MetS affects a significant percentage of the Asian and European populations. According to Ranasinghe et al., the estimated ranges of MetS are 11.9–37.1% in Asia and 11.6–26.3% in Europe [5]. In South Asia, Aryal et al. estimated the prevalence of MetS to be between 14.0% and 32.5% [6]. Bangladesh, a developing country in South Asia, faces the burden of NCD risk factors, as reported by Riaz et al. [7]. Bangladesh has experienced rapid urbanisation over the past few decades because of its economic growth [8, 9]. However, this economic growth and urbanisation have led to concerns about a potential increase in chronic diseases because of the adoption of a sedentary lifestyle. A study by Gupta et al. reported the prevalence of MetS as 15.2% and 16.6% in Bangladeshi adults following NCEP ATP III and IDF criteria, respectively [10]. On the contrary, a systematic review and meta‐analysis by Chowdhury et al.'s used NCEP ATP III criteria and found that 37.0% of the Bangladeshi population is affected by MetS [11], which is a pressing public health issue that requires immediate attention.

In the last decade, cases of cardiovascular disease have risen steadily in Bangladesh [12], and it is now one of the leading causes of death. This is expected to continue as the duration of the disease and related disabilities increase, resulting in a greater socio‐economic burden. To address this issue, it is crucial to prioritise the prevention of and reduction in MetS. To effectively combat MetS, it is essential to have current estimates of its prevalence and associated factors. Therefore, it is important to survey the prevalence of MetS in Bangladesh and gain a preliminary understanding of relevant risk and protective factors. This study aimed to provide current information on the prevalence and associated factors of MetS among Bangladeshi adults over 18 years old. Additionally, this study estimated the prevalence of MetS on the basis of gender and different groups, including healthy individuals, those with diabetes and those with hypertension.

## 2 Methods

### 2.1 Study Area and Population

The research was carried out between January 2019 and December 2021 at the Department of Biochemistry and Molecular Biology at Shahjalal University of Science and Technology (SUST) in Sylhet, Bangladesh. For this study, we used a simple random sampling technique to select participants from the Sylhet region (urban and suburban areas) in Bangladesh. Over 1000 individuals were invited, and 660 agreed to participate. After excluding seven individuals, we included 653 participants in the study. We divided the participants into three groups: diabetic, hypertensive and healthy control. For the diabetic group, we enrolled some of the participants from the Sylhet Diabetic Hospital, who were there for their regular health check‐ups. To be included in the study, participants had to be (i) 18 years or older, (ii) willing to participate and (iii) of any gender. Participants were excluded if they had (i) chronic inflammation, (ii) liver or kidney dysfunction or any infectious diseases, (iii) were pregnant or breastfeeding or (iv) didn't complete the questionnaire or tests. The Ethical Review Committee at the Biochemistry and Molecular Biology Department, SUST, approved the survey with the approval code 02/BMB/2019. Before the study began, written informed consent was obtained from all participants. All methods of the study followed relevant guidelines and regulations.

### 2.2 Data Collection

The supervisor/team leader provided training to the Master's thesis students and research technicians on their respective roles in the interview data collection process. Then, they conducted interviews with study participants and collected their anthropometric and demographic information. The height, weight, waist and hip circumference (WC and HC) of the participants were measured using standard procedures described elsewhere in detail [13, 14, 15, 16, 17, 18, 19]. In brief, the participants' body weight was measured using modern electronic digital LCD weighing scales (Beurer 700, Germany) to the nearest 0.1 kg. Height was measured to the nearest 0.1 cm using a height‐measuring tape while the participants stood erect. The body mass index (BMI) was calculated as weight in kilogram divided by height in metre squared. To measure WC, a general tape was used. It was placed midway between the lower margin of the last palpable rib and the iliac crest on the mid‐axillary line. HC was measured at the largest circumference of the buttocks. Blood pressure was measured with a standardised automatic sphygmomanometer (Omron M10; Omron Corporation, Tokyo, Japan). The study participants rested for at least 10 min before three consecutive blood pressure readings were taken, with 5 min between each reading. To prevent errors, the first blood pressure reading was disregarded, and the average of the second and third readings was used to determine the systolic and diastolic blood pressures (SBP and DBP, respectively). The participant's lifestyle information and disease history were also included in the questionnaire. After measuring height, weight and blood pressure, we provided participants with information regarding their BMI and blood pressure status. The information was conveyed through health messages in Bengali, the local language. These messages included details about the risk factors associated with obesity and hypertension, such as lack of physical activity, uncontrolled blood pressure, unhealthy diet, poor lifestyle choices and smoking. We advised participants with MetS to seek guidance from the health education sector.

### 2.3 Sampling and Laboratory Analyses

The participants provided venous blood samples in the morning after fasting overnight. The serum was extracted from the samples by centrifugation and stored at a temperature of −80°C until the markers could be analysed. Enzymatic colorimetric methods were deployed to measure the levels of serum glucose, creatinine, total cholesterol (TC), triglycerides (TG), low‐density lipoprotein cholesterol (LDL‐C) and high‐density lipoprotein cholesterol (HDL‐C). All the biochemical markers were measured using a semi‐automated bioanalyzer (Humalyzer 3000; USA) [20, 21, 22]. The biochemical measurements were carried out in a single laboratory, using consistent methods throughout the assay period.

### 2.4 Diagnostics Criteria

In order to diagnose MetS, the National Cholesterol Education Program—Adult Treatment Panel III (NCEP‐ATP III) criteria were used, which require a minimum of three of the following components [23]: a waist circumference of 90 cm or more in men, or 80 cm or more in women; triglyceride (TG) levels of 150 mg/dL or more; high‐density lipoprotein cholesterol (HDL‐C) levels of <40 mg/dL in men, or <50 mg/dL in women; blood pressure readings of 130/85 mmHg or more, or taking antihypertensive medication; fasting blood glucose (FBG) levels of 110 mg/dL or more, or taking antidiabetic medications. The definition of physical activity was based on the Global Physical Activity Questionnaire (GPAQ) created by the World Health Organization (WHO) [24]. It was classified as low, moderate or vigorous on the basis of the level of exertion involved in the task. Examples of low activity include office jobs and light housework, moderate activity includes general walking, swimming and light cleaning, whereas vigorous activity includes lifting, carrying, jogging and sports [25].

### 2.5 Statistical Analyses

Continuous variables are presented as mean values and categorical variables as proportions. An independent sample *t*‐test was used for continuous variables, and a chi‐squared test was used for categorical variables to determine differences between groups. A multivariate logistic regression analysis was conducted to test risk factors associated with the odds of MetS. MetS and its components were dependent variables in this regression model, whereas baseline parameters, anthropometrics, demographics and lifestyles were independent variables. *P* values of less than 0.05 were considered statistically significant. The statistical analyses were performed using SPSS version 25.0 (IBM, Chicago, IL, USA).

## 3 Results

### 3.1 Characteristics of the Participants

This study included 653 adults, consisting of 470 males and 183 females, with an average age of 39.4 ± 13.4 years (ranging from 18 to 85 years old). The characteristics of these participants are listed in Table 1. The average BMI of the participants was 24.6 ± 3.7 kg/m^2^, with no significant difference observed between genders. However, males had significantly higher average SBP, TG and creatinine levels than females (*p* < 0.05 for all cases). Conversely, females had significantly higher average values of FBG, TC, HDL‐C and LDL‐C (*p* < 0.05 for all cases). The participants were categorised into the healthy control, hypertensive or diabetic group on the basis of their BP and FBG levels, with a 17.2% prevalence of hypertension and a 15.6% prevalence of diabetes. Additionally, 21.9% of participants had both hypertension and diabetes. Approximately 21% of participants were smokers, whereas only 10.1% engaged in adequate physical activity.

**TABLE 1:** Baseline characteristics of the participants by sex.

	Overall	Male	Female	*p*
*N*	653	470	183	—
Age, year	39.4 ± 13.4	39.5 ± 13.2	39.2 ± 13.9	0.834
WC, cm	85.0 ± 10.9	86.1 ± 10.9	82.4 ± 10.8	0.001
HC, cm	92.1 ± 8.5	92.4 ± 8.4	91.5 ± 8.7	0.313
BMI, kg/m^2^	24.6 ± 3.7	24.6 ± 3.4	24.4 ± 4.5	0.618
SBP, mmHg	126.2 ± 15.5	127.0 ± 14.6	123.9 ± 17.6	0.031
DBP, mmHg	82.1 ± 10.0	82.5 ± 9.8	80.9 ± 10.7	0.084
FBG, mg/dL	7.0 ± 3.7	6.7 ± 3.5	7.8 ± 4.1	0.002
TG, mg/dL	188.0 ± 115.2	194.1 ± 111.1	171.1 ± 124.7	0.039
TC, mg/dL	202.5 ± 74.7	198.5 ± 70.3	215.3 ± 86.7	0.035
HDL‐C, mg/dL	33.5 ± 12.3	32.6 ± 12.7	36.6 ± 10.1	0.002
LDL‐C, mg/dL	134.2 ± 67.4	129.0 ± 60.8	151.0 ± 83.7	0.002
Creatinine, mg/dL	0.92 ± 0.27	0.98 ± 0.28	0.72 ± 0.16	0.000
Hypertension (%)	17.2	20.0	9.4	0.002
Diabetes (%)	15.6	13.3	22.0	0.009
Hypertensive diabetic (%)	21.9	18.7	30.8	0.002
Smoking (%)				0.000
No	78.8	71.2	100.0	
Yes	21.2	28.8	0.0	
Physical activity (%)				0.008
Low	22.4	18.5	31.6	
Moderate	67.5	70.3	60.9	
Adequate	10.1	11.2	7.5	

*Note*: Values are presented as mean ± SD for continuous variable, and percentage for categorical variables. *p* values are obtained from the independent sample *t*‐test and the chi‐squared test.

Abbreviations: BMI, body mass index; DBP, diastolic blood pressure; FBG, fasting blood glucose; HC, hip circumference; HDL‐C, high‐density lipoprotein cholesterol; LDL‐C, low‐density lipoprotein cholesterol; SBP, systolic blood pressure; TC, total cholesterol; TG, triglycerides; WC, waist circumference.

### 3.2 Prevalence of MetS by Genders and Health Status

**FIGURE 1:**
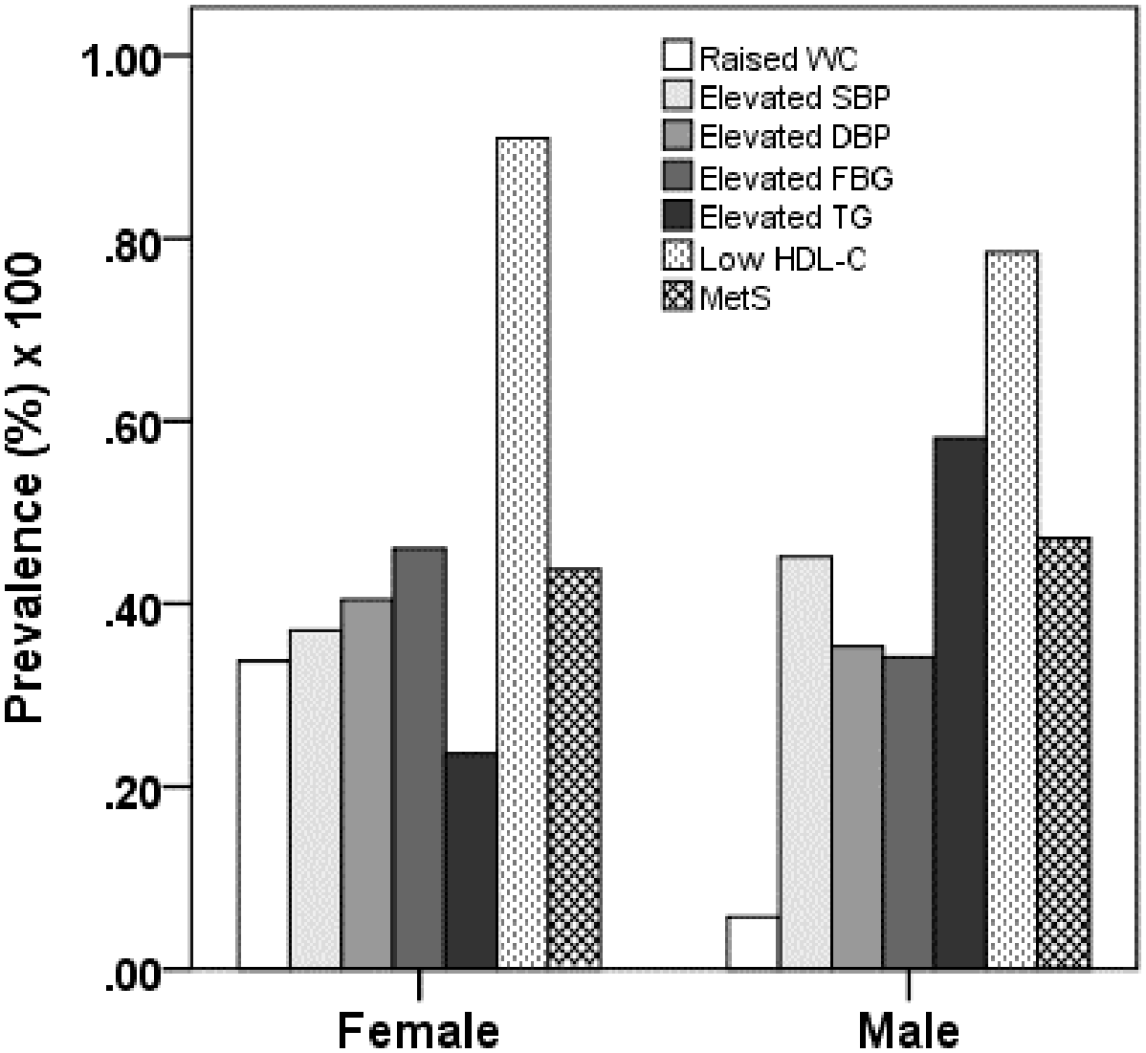
Prevalence of MetS and its components by the sex groups. *p* < 0.001 when the prevalence of elevated FBG and elevated TG are compared between the sex groups. *p* < 0.05 when the prevalence of low HDL‐C is compared between the sex groups. *p* values are obtained from the chi‐squared test.

The study found that the overall prevalence of MetS was 45.5%, with no significant difference between males (45%) and females (45.9%) (Table 2). However, there were differences in the prevalence of the components of MetS between the genders (Figure 1). The prevalence of MetS in the healthy control, hypertensive and diabetic groups was 19.7%, 70.2% and 46.8%, respectively (Table 2). Individuals who were both hypertensive and diabetic had a higher prevalence of MetS (77.3%) than those in other groups.

**TABLE 2:** Prevalence of MetS in different groups.

	*N*			MetS (%)			
	Overall	Male	Female	Overall	Male	Female	*p*
Healthy control	290	223	67	19.7	22.0	11.7	0.076
Hypertensive	118	95	23	70.2	69.7	73.3	0.774
Diabetic	102	64	38	46.8	55.9	31.4	0.021
Hypertensive diabetic	143	88	55	77.3	69.9	89.8	0.008
Overall	653	470	183	45.5	45.0	45.9	0.851

*Note*: Healthy control: participants who had no hypertension and diabetes. Hypertensive diabetic: participants who had both hypertension and diabetes. *p* values are derived from the chi‐squared test.

### 3.3 Prevalence of MetS and its Components by Age Groups

Table 3 presents the age‐stratified prevalence of MetS and its components, with the highest prevalence of MetS observed in the 46–55 years age group in males (68%) and >55 years age group in females (73.9%). There was an increased trend in the prevalence of MetS and its components (except WC in males) with age in both males and females (*p* < 0.01, at least for all cases). Both genders had the highest SBP and FBG levels in the >55 year age group. The most common component of MetS was low levels of HDL‐C, which affected more than 80% of the sample.

**TABLE 3:** Prevalence of MetS and its components in different age groups.

Parameters	Age group (years)					*p*	Total
	≤25	26–35	36–45	46–55	>55		
Overall							
MetS	25.5	39.3	64.6	66.9	66.2	0.000	45.5
Male							
MetS	29.3	34.1	67.0	68.0	62.7	0.000	45.0
Raised WC	3.0	8.6	2.4	3.5	10.4	0.204	5.4
Elevated SBP	34.3	29.8	48.2	59.8	66.7	0.000	46.3
Elevated DBP	31.3	22.6	68.8	62.9	56.9	0.000	49.0
Elevated FBG	8.1	25.9	42.9	59.4	78.4	0.000	39.5
Elevated TG	41.1	61.3	74.0	62.2	48.8	0.000	59.0
Low HDL‐C	85.6	84.0	84.9	84.6	54.5	0.001	82.1
Female							
MetS	16.7	55.6	58.7	61.9	73.9	0.000	45.9
Raised WC	14.3	29.2	56.7	6.7	40.9	0.000	30.1
Elevated SBP	19.0	33.3	47.8	57.1	73.9	0.000	42.8
Elevated DBP	19.0	44.4	52.2	57.1	69.6	0.000	45.3
Elevated FBG	14.6	55.6	77.3	76.2	95.7	0.000	59.6
Elevated TG	12.5	44.0	41.0	70.0	66.7	0.000	41.4
Low HDL‐C	95.0	100.0	84.8	90.0	83.3	0.257	91.3

*Note*: *p* values are obtained from the chi‐squared test.

Abbreviations: DBP, diastolic blood pressure; FBG, fasting blood glucose; HDL‐C, high‐density lipoprotein cholesterol; SBP, systolic blood pressure; TG, triglycerides; WC, waist circumference.

### 3.4 Logistic Regression to Identify Risk Factors Associated With MetS

According to the results of multivariate logistic models (as shown in Table 4), the most significant risk factor for MetS in both genders was found to be age over 36 years (*p* < 0.05 for all cases). Additionally, an increased BMI was independently and significantly associated with a higher risk of MetS (*p* < 0.05 for all cases). Hypertension and diabetes also were associated with an increased risk of MetS, even after adjusting for age and sex (*p* < 0.05 for all cases). However, no significant association was observed between physical activity and the participants' risk of MetS.

**TABLE 4:** Risk factors associated with MetS in participants by multivariate regression analysis.

Risk factors	Overall		Male		Female	
	OR (95% CI)	*p*	OR (95% CI)	*p*	OR (95% CI)	*p*
Age						
≤25	Reference		Reference		Reference	
26–35	1.48 (0.85–2.59)	0.168	1.07 (0.56–2.05)	0.826	4.32 (1.32–14.15)	0.016
36–45	4.02 (2.39–6.76)	0.000	3.98 (2.18–7.28)	0.000	4.00 (1.37–11.67)	0.011
46–55	4.86 (2.78–8.50)	0.000	4.59 (2.45–8.61)	0.000	5.03 (1.43–17.63)	0.012
>55	5.37 (2.87–10.02)	0.000	4.04 (1.95–8.35)	0.000	13.40 (3.67–48.87)	0.000
BMI						
Normal	Reference		Reference		Reference	
Overweight	1.52 (1.02–2.27)	0.039	1.32 (0.84–2.10)	0.229	2.97 (1.24–7.12)	0.014
Obesity	4.23 (2.44–7.32)	0.000	4.02 (2.09–7.74)	0.000	4.72 (1.66–13.45)	0.004
Hypertension						
No	Reference		Reference		Reference	
Yes	16.13 (10.25–25.38)	0.000	10.08 (5.83–17.43)	0.000	54.00 (15.03–194.06)	0.000
Diabetes						
No	Reference		Reference		Reference	
Yes	5.62 (3.82–8.25)	0.000	4.96 (2.67–9.21)	0.000	2.51 (1.03–6.10)	0.042
Physical activity						
High	Reference		Reference		Reference	
Moderate	1.36 (0.67–2.78)	0.394	1.00 (0.40–2.47)	0.999	0.80 (0.19–3.40)	0.766
Low	1.07 (0.57–2.02)	0.824	1.11 (0.52–2.37)	0.790	0.54 (0.14–2.11)	0.374

*Note*: Multivariate logistic regression was applied to evaluate the relationship between MetS and associated factors. Values are presented as OR (95% CI).

Abbreviations: CI, confidence interval; OR, odds ratio.

## 4 Discussion

This study was conducted to determine the prevalence of and risk factors associated with MetS among adults in Bangladesh. The overall prevalence of MetS was 45.5%, with no significant difference between the gender groups. The participants with hypertension and diabetes had the highest prevalence of MetS. We observed an increased trend in the prevalence of MetS and its components (except WC in males) with age in both genders. Of the MetS components, HDL‐C was the most prevalent form, which affected over 80% of the samples. Factors significantly associated with MetS in both genders were increased age, BMI, hypertension and diabetes.

Our study found a higher prevalence of MetS than estimates from previous studies. Chowdhury et al. reported a prevalence of 37% among the Bangladeshi population using NCEP ATP III criteria [11], whereas Gupta et al. reported lower prevalence rates of 15.2% and 16.6% following NCEP ATP III and IDF criteria, respectively [10]. This difference might be because, in the present study, we also enrolled diabetic (15.6%) and hypertensive (17.2%) individuals in addition to healthy individuals. In contrast, Chowdhury et al. utilised studies carried out at the local/community level to obtain the estimate [11]. On the contrary, Gupta et al. considered participants from rural and urban areas and did not differentiate between diabetic or hypertensive individuals [10].

Furthermore, a major portion of our study participants were used to living in suburban or urban areas, which might also influence the overall prevalence of MetS. In Gupta et al. study, a higher prevalence of MetS was found in urban residents than in rural residents in Bangladesh [10]. Similarly, an increased prevalence of MetS was found in the urban population in India, a neighbouring country of Bangladesh [26]. The behavioural issues, comparatively fatty food habits and obesogenic environment may contribute to the higher risks of having MetS among the urban population. A recent review indicated that around 25% of the adult population in India has MetS, with a higher prevalence in women than in men [27]. A report from another neighbouring country, Pakistan, showed the prevalence of MetS ranging from 18% to 46% [28]. A further study reported the crude prevalence of MS at 27.1% and age‐adjusted prevalence at 24.3%, with higher in women than in men in Sri Lanka, and this prevalence was significantly higher among urban adults than rural adults [29]. However, the prevalence of MetS may vary depending on the criteria used for defining it in the study.

In the current study, we observed that older age is associated with MetS in both genders. Upon analysing various age groups, we observed that MetS was relatively less prevalent in males under the age of 36 and females under the age of 26 as compared to older individuals. This implies that the metabolic system may become weaker with age. Furthermore, a previous study has indicated that regular physical activity can help in enhancing blood pressure and lipid levels to achieve optimal levels [30]. Over 7 years, a study was conducted on older women in Central Europe, revealing that physical activity considerably decreases the chances of MetS [31]. Additionally, Lee, Kim and Jeon (2016) demonstrated that having intense physical activity six times a week decreased the likelihood of developing MetS by 35% [32]. Increased BMI was associated with MetS in our study, similar to previous studies [10, 11]. Furthermore, both hypertension and diabetes showed an independent association with MetS in our study. Of the MetS components, low HDL‐C was the most prevalent form in students and staff. The findings suggest these groups are at increased risk of cardiovascular disease. In individuals who are obese, adipose cells release free fatty acids and cytokines, such as tumour necrosis factor‐alpha (TNF‐α). These substances block signal transduction pathways that depend on phosphatidylinositide‐3‐kinase, which results in reduced glucose uptake in the liver and skeletal muscles [3, 33]. This forces pancreatic β‐cells to secrete excessive insulin, leading to hyperglycaemia or diabetes [34]. As we age, blood vessels gradually lose elasticity and gain resistance, slowing blood flow down. Poor circulation causes lipids to accumulate in the abdomen and release free fatty acids into the serum, resulting in increased insulin resistance and elevated serum triglycerides [35]. These physiological and biological processes along with increased adiposity increase the risk of MetS. This study shows that the prevalence of MetS and its components mostly increases with age, and its associated factors, like diabetes and hypertension.

We found a slightly higher prevalence of MetS in females than in males, which is close to the findings of previous studies [10, 11]. In most country‐wide estimates, MetS was more prevalent in women than men [36]. Various factors could explain the differences between men and women in terms of MetS prevalence. For one, women who have gone through menopause are more likely to have central obesity and insulin resistance, which could contribute to the disparity [37]. Our research found that obesity is a significant risk factor for MetS. Other studies have also shown that pregnant women are more likely to be obese, and multiple pregnancies can further increase the risk of developing MetS [38]. Although gender and age cannot be changed and are considered risk factors for MetS, there are other risk factors that can be modified, such as lifestyle behaviours. These behaviours can provide more practical and relevant interpretations in real‐life situations. One such behaviour that can significantly increase the risk of MetS is having sedentary work. As MetS is common among Bangladeshis, it is important to conduct specific research on the causes, spread and treatment of MetS in our country. This research should include basic, clinical and epidemiological aspects.

The study's major strength was using a clear case definition that relied on diagnostic confirmation through blood tests to identify individuals with dyslipidaemia and diabetes. Another strong point was the inclusion of several factors in the analysis that may increase the risk of MetS. However, the study also had some limitations. First, because of its cross‐sectional nature, it was not possible to establish a causal relationship between risk factors and MetS. Second, relatively a small number of participants enrolled in the study; therefore, the findings may not be applicable to the entire population of Bangladesh. To gain a better understanding of the prevalence of MetS in both rural and urban adults, additional studies are required. Furthermore, the prevalence of MetS was based on a single measurement of blood parameters, which may have led to minor inaccuracies.

## 5 Conclusion

MetS is common among Bangladeshi adults. The most widespread component of MetS was low levels of HDL‐C, which was present in over 80% of the samples. Age, BMI, hypertension and diabetes were significant factors contributing to MetS in both genders. The study results indicate that MetS has become an important health concern for the Bangladeshi population. Therefore, it is essential to implement preventive healthcare strategies urgently to reduce the burden of MetS and related morbidity and mortality. Multidisciplinary and multi‐sectoral preventive measures at individual, community and societal levels are required to combat the growing epidemic of MetS in Bangladesh. The focus should be on promoting healthy food habits and physically active lifestyles.

## Author Contributions

N.A. conceptualised the study, designed the research, interpreted the results, and drafted and revised the manuscript. A.T., A.D.T., N.J.K. and K.A.F. assisted with sampling and experiments. F.I. contributed to review and editing of the manuscript. All authors have read and approved the final version of the manuscript.

## Ethics Statement

This study was approved by the Internal Ethics Committee of the Department of Biochemistry and Molecular Biology, Shahjalal University of Science and Technology, Sylhet 3114, Bangladesh (reference no. 02/BMB/2019). All methods of the study were carried out in accordance with relevant guidelines and regulations. Informed consent was obtained from all participants before inclusion in the study.

## Conflicts of Interest

The authors declare no conflicts of interest.

## Data Availability

The datasets used and/or analysed during the current study are available from the corresponding author upon reasonable request.
